# Attenuated Tonic and Enhanced Phasic Release of Dopamine in Attention Deficit Hyperactivity Disorder

**DOI:** 10.1371/journal.pone.0137326

**Published:** 2015-09-30

**Authors:** Rajendra D. Badgaiyan, Sampada Sinha, Munawwar Sajjad, David S. Wack

**Affiliations:** 1 Molecular and Functional Imaging Laboratory, Department of Psychiatry, University of Minnesota, Minneapolis, Minnesota, United States of America; 2 Neuromodulation Program, University of Minnesota Twin City Campus, Minneapolis, Minnesota, United States of America; 3 Laboratory of Advanced Radiochemistry, University of Minnesota Twin City Campus, Minneapolis, Minnesota, United States of America; 4 Department of Nuclear Medicine, University at Buffalo, Buffalo, New York, United States of America; Queens College and the Graduate Center, CUNY, UNITED STATES

## Abstract

It is unclear whether attention deficit hyperactive disorder (ADHD) is a hypodopaminergic or hyperdopaminergic condition. Different sets of data suggest either hyperactive or hypoactive dopamine system. Since indirect methods used in earlier studies have arrived at contradictory conclusions, we directly measured the tonic and phasic release of dopamine in ADHD volunteers. The tonic release in ADHD and healthy control volunteers was measured and compared using dynamic molecular imaging technique. The phasic release during performance of Eriksen’s flanker task was measured in the two groups using single scan dynamic molecular imaging technique. In these experiments volunteers were positioned in a positron emission tomography (PET) camera and administered a dopamine receptor ligand ^11^C-raclopride intravenously. After the injection PET data were acquired dynamically while volunteers either stayed still (tonic release experiments) or performed the flanker task (phasic release experiments). PET data were analyzed to measure dynamic changes in ligand binding potential (BP) and other receptor kinetic parameters. The analysis revealed that at rest the ligand BP was significantly higher in the right caudate of ADHD volunteers suggesting reduced tonic release. During task performance significantly lower ligand BP was observed in the same area, indicating increased phasic release. In ADHD tonic release of dopamine is attenuated and the phasic release is enhanced in the right caudate. By characterizing the nature of dysregulated dopamine neurotransmission in ADHD, the results explain earlier findings of reduced or increased dopaminergic activity.

## Introduction

Attention deficit hyperactivity disorder (ADHD) is the most common psychiatric condition of childhood [1]. Converging evidence from clinical, neuroimaging and animal studies indicates that dopamine neurotransmission is dysregulated in this disorder [2, 3]. It is however unclear whether ADHD is a hypodopaminergic or hyperdopaminergic condition because earlier studies have arrived at contradictory conclusions [3]. Findings suggesting reduced dopaminergic activity include small volume of the caudate [4–7], reduced regional blood flow in the striatum [8], increased uptake of ^18^F-DOPA [9], attenuation of methylphenidate induced dopamine release in the caudate [10], correlation of methylphenidate induced dopamine release with clinical symptoms [11], reduced dopamine synaptic markers [12], and clinical efficacy of dopaminergic agents [13]. Some of the data acquired in laboratory animals are also consistent with the above findings. Thus, increase in spontaneous motor activity after lesions of dopaminergic neurons in rats and reduced attention span in D_2_ knockout mice [14] indicate that symptoms of inattention and hyperactivity are elicited under hypodopaminergic condition [15].

Another set of data however contradicts the hypodopaminergic concept and suggests that ADHD could be a hyperdopaminergic condition. For example, finding of a positive correlation between levels of dopamine metabolites in the CSF and clinical symptoms of ADHD patients [16] is inconsistent with the concept of reduced dopaminergic activity. The finding of greater d-amphetamine-induced reduction in the ligand BP in ADHD volunteers as compared to healthy control suggests that ADHD may be hyperdopaminergic condition [17]. Reports of hyperactivity and inattention in genetically engineered hyperdopaminergic mice [18, 19] also support the hyperdopaminergic concept. Further, if dopamine is hypoactive then all dopaminergic agents should be clinically effective. Indeed some of these agents are used as the first line treatment, but not all dopaminergic medications have clinical efficacy. For example, levodopa does not improve clinical symptoms of ADHD when administered either alone or in combination with carbidopa [20]. These findings have prompted many investigators to suggest that ADHD is a hyperdopaminergic condition [16], particularly because increased dopaminergic activity is known to induce motor hyperactivity and impairment of inhibitory control [21].

Thus, different sets of data indicate either hypoactive or hyperactive dopamine system in ADHD. The reason for contradictory findings is unclear but it could be due to use of indirect evidence to estimate dopaminergic activity. These estimates do not provide reliable information on the tonic (dopamine release at rest) and phasic (dopamine release during task performance) release of dopamine in the brain. Based on the available data we hypothesized that in ADHD there is attenuation of the tonic and enhancement of phasic release of dopamine. To examine validity of this hypothesis in this study we measured tonic release of dopamine at rest and the phasic release during performance of a modified Eriksen’s flanker task in adult ADHD and healthy control volunteers. We used recently developed single scan dynamic molecular imaging technique [22–32] for detection of dopamine release in the brain. The technique allows detection and mapping of dopamine released acutely at rest or during task performance. Separate measurement of the tonic and phasic release of dopamine will allow better understanding of the status of dopamine neurotransmission in ADHD.

## Methods

This study was approved by Partners IRB Boston, MA 02114 and IRB of the University at Buffalo, NY. All volunteers provided written informed consent approved by the institutional review board.

The study consists of separate experiments designed to study either phasic or tonic release of dopamine. A total of 22 adult ADHD and 22 healthy control volunteers of either sex participated in these experiments. All volunteers were right handed by the criteria included in Edinburgh handedness inventory. Healthy control volunteers had no personal or family history of a psychiatric or neurological condition, no history of substance abuse/dependence, and no significant cognitive deficit. Psychopathology in both healthy control and ADHD volunteers was evaluated using Structured Clinical Interview for DSM-IV (SCID) [33] and neurological deficits were excluded by clinical examination. Cognitive deficits were evaluated using the Mini Mental State Examination (MMSE). A urine toxicological screen was performed on all volunteers on the day of study to exclude substance use. The ADHD and healthy control volunteers had similar group means of age, sex, race, and MMSE score. The study did not include pregnant women and volunteers under the age of 18 years because the risk of exposure to ionizing radiation is unknown in unborn babies and children. A urine pregnancy test was performed on all female volunteers on the day of the study to exclude pregnancy. Volunteers who had a history of comorbid Axis I diagnosis, claustrophobia, or significant prior radiation exposure were also excluded. Use of a dopamine altering medication in the last six months was one of the exclusion criteria. All volunteers provided written informed consent approved by the institutional review board.

### Assessment Measures and Experimental Procedures

As mentioned above, the psychopathology, cognitive deficit, and neurological disorder were excluded using the SCID, MMSE and clinical neurological examination. The SCID was modified to include questions derived from Kiddie-SADS-E [34] to ensure inclusion of childhood psychiatric diagnosis. Diagnosis of ADHD was made both at the present time (last one month) and over the lifetime and the severity of symptoms was assessed using ADHD Rating Scale [35]. Before ADHD was diagnosed, it was ensured that volunteers meet full DSM-IV criteria for combined subtype by the age of seven as well as within the past month; they describe a chronic course of ADHD symptomatology; and endorse a moderate or severe level of impairment due to these symptoms.

On the day of study volunteers were positioned on the bed of positron emission tomography (PET) camera and administered a single intravenous bolus of a radiolabeled dopamine receptor ligand ^11^C-raclopride (mean injected dose 14.47±0.88 mCi) at high specific activity (mean specific activity 1.76±0.65 Ci/μMol). After the injection PET data were acquired dynamically in list mode.

In experiments on tonic release 10 adult ADHD (mean age 24.7±4.9) and 12 healthy control volunteers of either sex (mean age 22.7±2.4 years) participated. These volunteers were positioned on the bed of positron emission tomography (PET) camera and administered a single intravenous bolus of a radiolabeled dopamine receptor ligand (^11^C-raclopride) at high specific activity (mean specific activity 2.60±1.94 Ci/μMol). Mean injected dose of the radioligand was 14.98±0.36 mCi. Immediately after the ligand injection PET data acquisition started and volunteers were asked to stay still in the scanner for 45 min.

The phasic release was studied in 11 adult ADHD (mean age 30.8±12.4) and 11 healthy control (mean age 33.1±8.3) volunteers. After positioned on the bed of PET camera, volunteers received a single intravenous bolus of the ligand ^11^C-raclopride (mean injected dose 14.47±0.88 mCi) at high specific activity (mean specific activity 1.76±0.65 Ci/μMol). After the injection PET data were acquired dynamically in list mode. During PET data acquisition volunteers were asked to perform a modified Eriksen’s flanker task under Congruent and Incongruent conditions [36]. In this task on a computer monitor volunteers were shown a series of 7 arrowheads pointing either to the left or right and asked to indicate direction of the arrowhead located at the center of the series (target arrowhead) by pressing a key on the keypad using the right index and middle fingers. They were required to respond as quickly and as accurately as possible. Each arrowhead was presented for 500 msec and inter-stimulus interval was set at 750 msec. Response time and accuracy were recorded in each trial. In the Congruent condition (which began 5 min after the ligand injection and continued for 20 min) both the target and flanker arrowheads pointed to the same direction, either left or right (<<<<<<< or >>>>>>>). These stimuli were presented pseudo randomly to ensure equal number of right and left-pointing trials. In the Incongruent condition (20 min duration) the target and flanker arrowheads pointed to different directions (<<<><<< or >>><>>>). Response execution in this condition required inhibition of the prepotent response indicated by a majority of stimuli (flanker arrowheads). Thus, inhibition of unwanted competing responses was required in the Incongruent but not in Congruent condition. We used this task because inability to inhibit unwanted responses is an important clinical feature of ADHD.

### Analysis of PET Data

In this study we dynamically measured values of the receptor kinetic parameters that describe ligand binding and displacement using the single scan dynamic molecular imaging technique [22–30]. The technique exploits the competition between ligand and endogenously released dopamine for occupancy of receptor sites. As a result of this competition the ligand is displaced from receptor sites and the displacement can be detected as rapid decline in PET count, if the ligand is radiolabeled. Changes in the PET count was analyzed using two receptor kinetic models: the linear extension of simplified reference region model (LE-SRRM) [37] and the extended simplified reference tissue model (E-SRTM)[38]. Reliability and sensitivity of the LE-SRRM has been validated in a series of experiments conducted in our laboratory [22–30] and elsewhere [31, 32]. The E-SRTM is relatively newer model, which allows measurement of the values of receptor kinetic parameters separately in each condition. We used this model to measure the values separately in the Congruent and Incongruent condition. In this study we used both models to measure values of receptor kinetic parameters because our earlier studies have shown that simultaneous use of the two models enhances reliability of data [27, 29, 30].

The PET data were preprocessed before using the receptor kinetic models. The preprocessing steps are described in details elsewhere [27, 37]. Briefly, the data were reconstructed as 128x128x63 element volumes with corrections for photon attenuation, random coincidences, scatter, and dead time. For movement correction all frames were realigned to the frame acquired at 25 min (reference frame). Thereafter a mean image of the frames acquired in the first 25 minutes of data acquisition was constructed. This image was used as the source image for spatial normalization with a raclopride template (based on MNI coordinates) developed in our laboratory. All frames were then smoothed using a 5 mm FWHM Gaussian filter. Routines of the statistical parametric mapping software (SPM8; Wellcome Department of Imaging Neuroscience, London) were used for some of these analyses. Thereafter, voxel-wise analysis was carried out on the realigned, normalized and smoothed images to estimate values of the receptor kinetic parameters in each volunteer. These values were then pooled across volunteers of each group (ADHD and healthy control) to obtain cohort mean. By comparing values measured in the Congruent and Incongruent conditions in each group, we localized the voxels where values changed significantly after task initiation (Incongruent condition). Thereafter we compared the values across groups to study changes in ADHD. The data were compared both in each voxel and also in each striatal region. The analysis involved some of the subroutines and procedures we previously developed in our laboratory to enhance accuracy and reliability [22, 23, 28, 30, 37, 39].

### Power Analysis

Because of low inter-subject variance, number of volunteers needed in each group in single scan dynamic molecular imaging technique is generally smaller than those used in other neuroimaging experiments. To arrive at this conclusion we analyzed published [22–30] and unpublished data acquired in our laboratory using this technique. The variance of the change in the rate of ligand displacement in these experiments was <0.03. Based on this variance, we estimated that data from 8 volunteers provide adequate power to arrive at a replicable conclusion at 95% confidence level even when the p-values are corrected for multiple comparisons. Other investigators that have used the technique have also arrived at a similar conclusion. Therefore, single scan dynamic molecular imaging studies conducted in our laboratory [22–30] and elsewhere [31, 32] have used 6 to 12 volunteers in each group. The data acquired in our previous experiments also suggested that dynamic molecular imaging data acquired from relatively small cohorts are reliable. In these experiments 6–10 volunteers participated and found phasic release of dopamine in the same areas where functional magnetic resonance imaging experiments reported increased activations in similar tasks [22, 23, 25–27, 40]. To further ensure reliability of results in the present experiment we examined the data of each ADHD and healthy control volunteer separately and found that the individual values are consistent with the mean cohort values.

## Results

**Fig 1:**
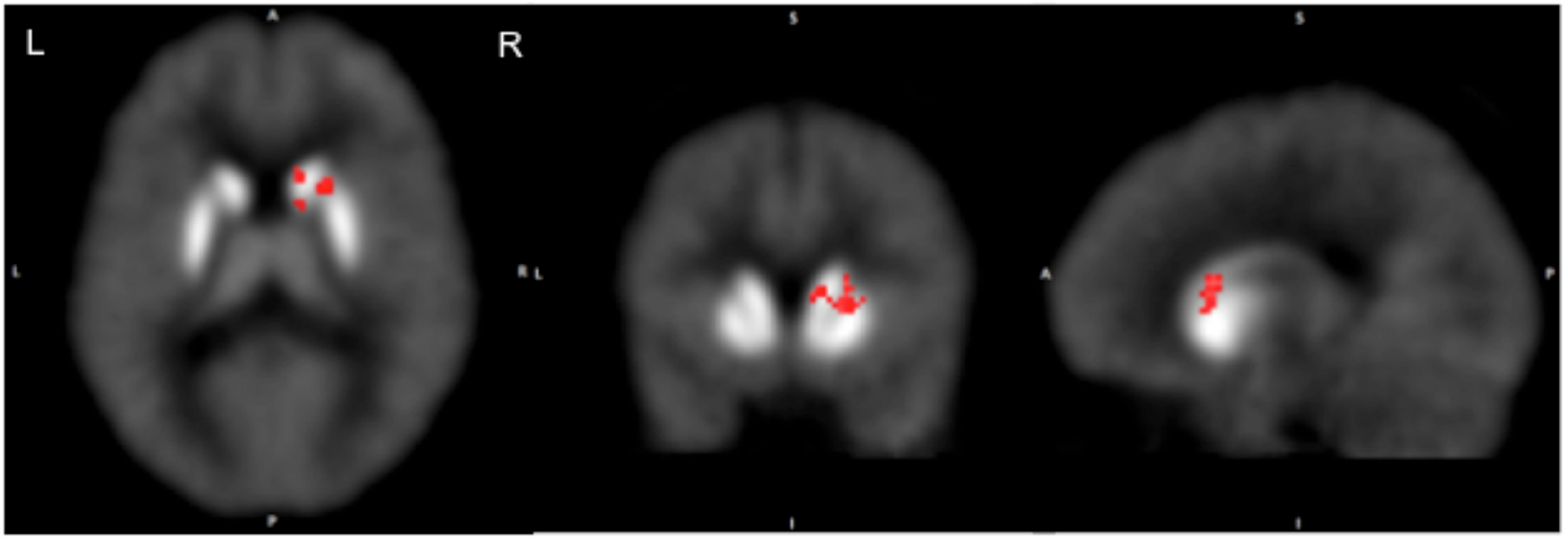
Comparison of the mean ligand binding potentials (BP) estimated at rest in ADHD and healthy control volunteers (ADHD>control). The BP was significantly greater in ADHD volunteers in the right caudate suggesting reduced tonic release of dopamine in this area.

To estimate the tonic release PET data acquired at rest were analyzed to measure values of the ligand BP in each voxel and in the caudate and putamen of each hemisphere separately. We found that the mean ligand BP in the right caudate of ADHD volunteers (3.19±0.23) was significantly higher (p<0.003; peak t = 9.02, confidence interval ±0.13 at 95% confidence level) than that of the healthy control (2.86±0.26; confidence interval ±0.15 at 95% confidence level) volunteers (**Fig 1**). It was higher in the other striatal regions also but the difference was not significant statistically (**Table 1**). The mean ligand BP of the entire striatum was 27% higher in the ADHD group (3.21±1.30) as compared to that in the healthy control group (2.53±0.85).

**Table 1:** MNI coordinates of the maximum values of the ligand BP measured at rest in ADHD and healthy control volunteers in the caudate and putamen.

	ADHD		Healthy Control	
	MNI (x,y,z)	BP	MNI (x,y,z)	BP
Right Caudate	14,18,6	4.31	14,18,4	3.47
Left Caudate	-14,18,6	3.40	-14,16,4	3.33
Right Putamen	26,0,4	4.91	28,-4,4	4.08
Left Putamen	-28,-10,4	4.73	-26,-8,4	4.05

Mean values of the other receptor kinetic parameters measured in the caudate of ADHD volunteers were also significantly different from the values measured in healthy control volunteers. Thus, the rate constant for ligand transfer (min^-1^) from free to the plasma compartment (k_2_) measured in the striatum was significantly lower (p = 0.029) in the ADHD group (mean 0.22±0.03) than the values obtained in healthy control group (mean 0.25±0.03). We also obtained significantly lower value (p = 0.029) of the ‘apparent’ rate constant for the ligand transfer (min^-1^) from the receptor to plasma (k_2a_) in ADHD group (mean 0.06±0.01) in comparison with the value estimated in the healthy control group (mean 0.07±0.01). Further, there was a significant reduction in the rate of ligand delivery to the striatum as compared to the rate in the reference region (cerebellum). The ratio of the rate (R_1_) in the ADHD group was 0.80±0.10 and 0.96±0.11 in healthy control group. The difference was significant statistically (p = 0.002). Mean values of k_2,_ k_2a_ and R_1_ were lower in the putamen and caudate of ADHD volunteers but the differences were statistically significant only for k_2a_ and R_1_.

The phasic release was studied by detecting and mapping dopamine released acutely during performance of a modified Eriksen’s flanker task under Congruent (control) and Incongruent conditions. We observed that ADHD and healthy control volunteers had similar levels of performance in the task. In the Congruent and Incongruent conditions ADHD volunteers made correct responses in 96.9±2.7% and 83.3±26.2% of trials respectively. Healthy control volunteers made 97±0.02% (Congruent) and 91.0±10.1% (Incongruent) correct response. Response times of ADHD and healthy control volunteers were also comparable. In the Congruent condition it was 600±101 (ADHD) and 602±151 msec (healthy control) and in the Incongruent condition the mean response time was 706±106 msec in ADHD and 695±183 msec in healthy control volunteers. Partial data of the phasic release in healthy control volunteers were reported in an earlier publication [27].

**Fig 2:**
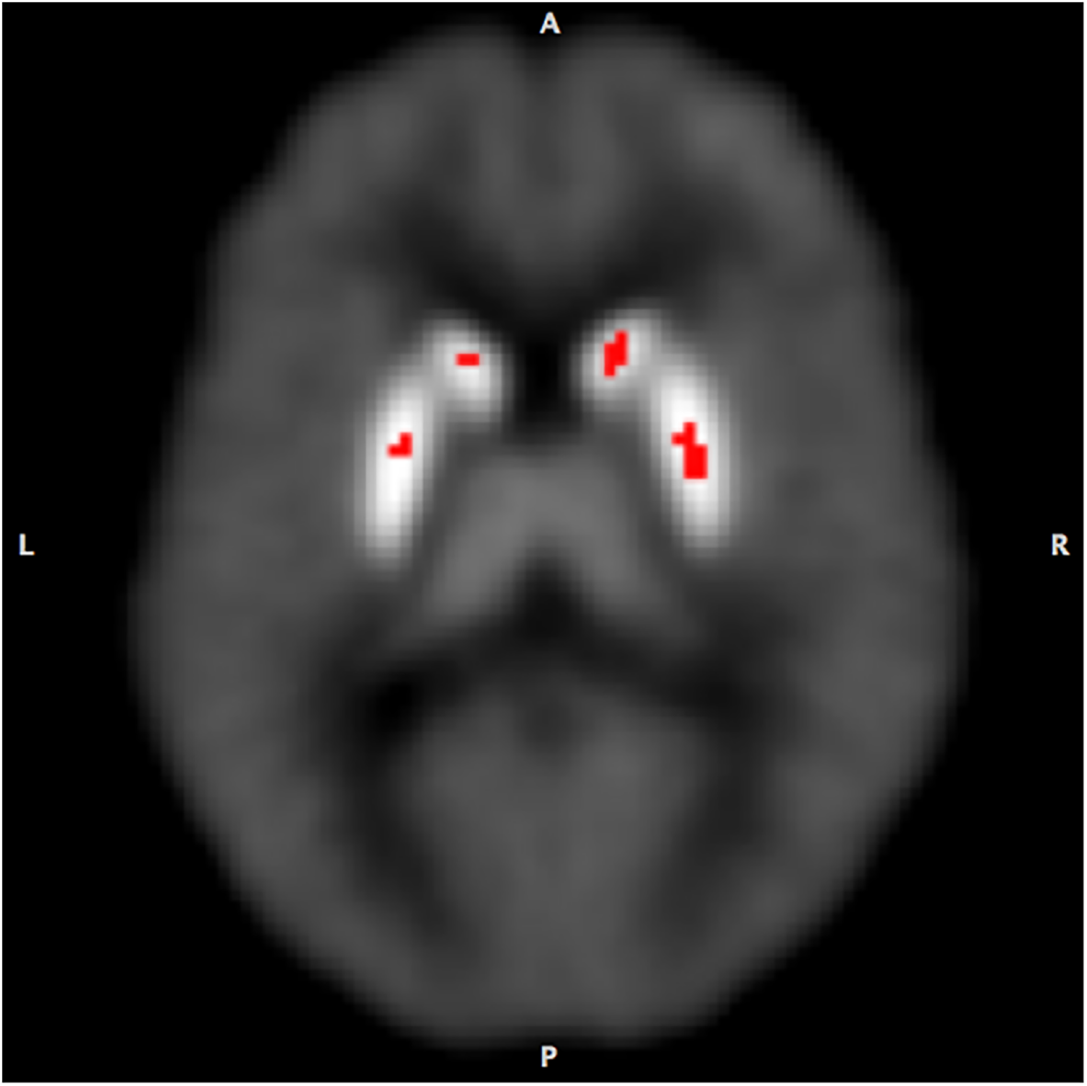
Increased ligand binding potential (BP) during task performance. In ADHD volunteers the ligand BP increased significantly in the caudate and putamen bilaterally in Incongruent condition which required inhibition of unwanted responses.

**Fig 3:**
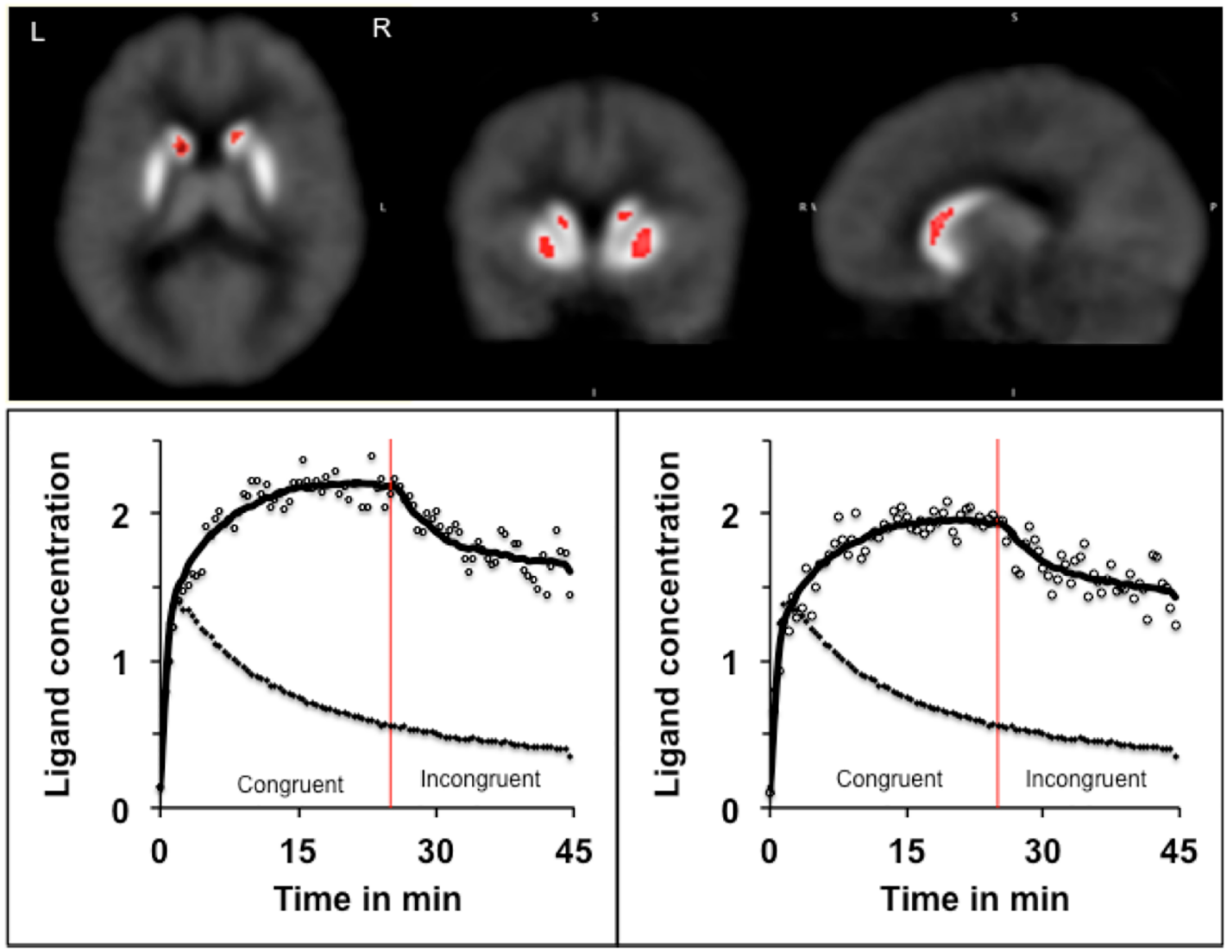
The rate of ligand displacement in the caudate in the Congruent and Incongruent conditions: Pictures show the location of right and left caudate where the rate of ligand displacement in the Incongruent condition was significantly greater than the rate in the Congruent condition, suggesting release of endogenous dopamine during task performance. The stereotactic coordinates (MNI) of the maxima were: 12, 14, 4 and -12, 14, 4. The curves show changes in the rate of ligand displacement over time during performance of the task under Congruent (control) and Incongruent conditions. The upper curves show the PET count (open circles) and the model fit (solid line). The lower curves (filled circle) depict the PET count in the reference region (cerebellum). Because of the lack of dopamine receptors in the cerebellum, the lower curve represents changes in nonspecific binding. Significant change after initiation of the Incongruent condition (red line) was observed in the striatum but not in the cerebellum. The ligand concentration is shown as x10, 000 mBq.

**Fig 4:**
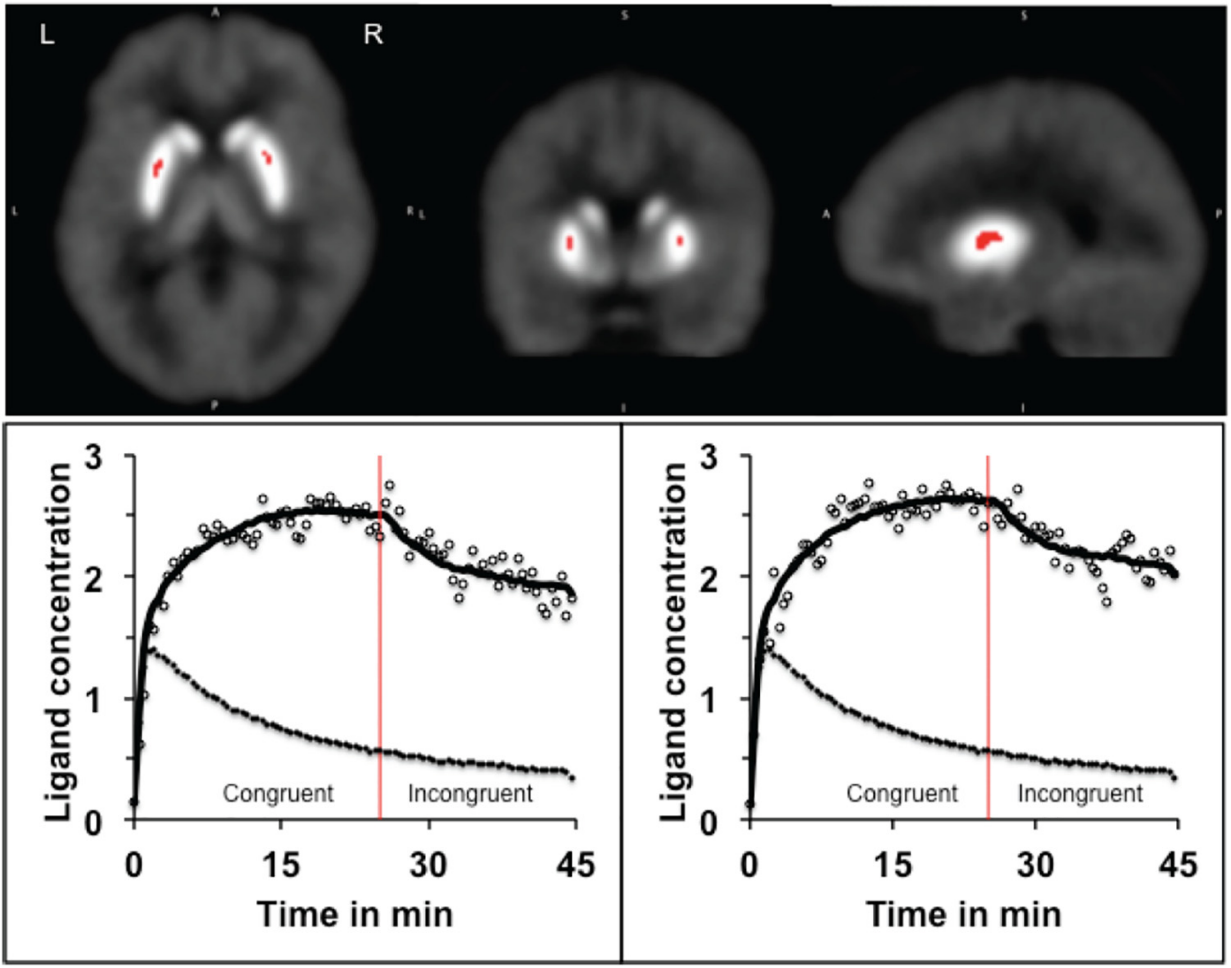
The rate of ligand displacement in the putamen in Congruent and Incongruent condition: Pictures show the location of right and left putamen where the rate of ligand displacement in the Incongruent condition was significantly greater than the rate in the Congruent (control) condition, suggesting endogenous dopamine release during task performance. The curves represent changes in the ligand concentration in the putamen and the reference region as explained in the legend for Fig 2.

**Fig 5:**
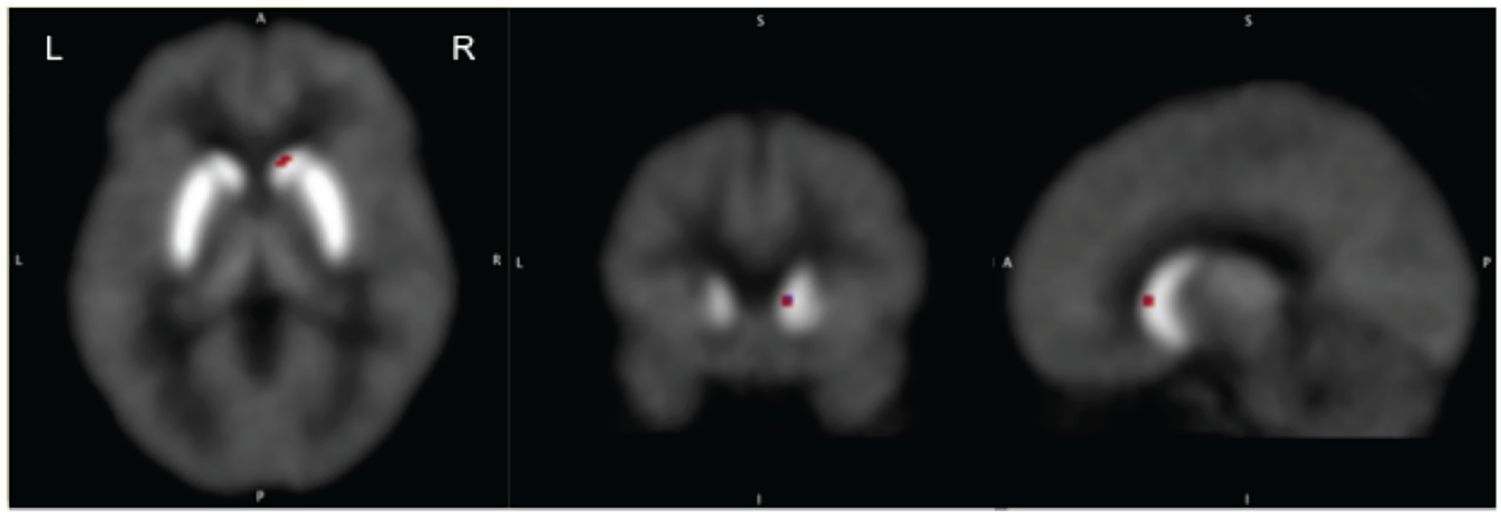
Comparison of the ligand BP in ADHD and healthy control volunteers in the Incongruent condition (healthy control >ADHD). The comparison revealed significantly higher BP in the right caudate of ADHD volunteers. The MNI coordinates of the maxima were, x,y,z = 12,16,6).

**Fig 6:**
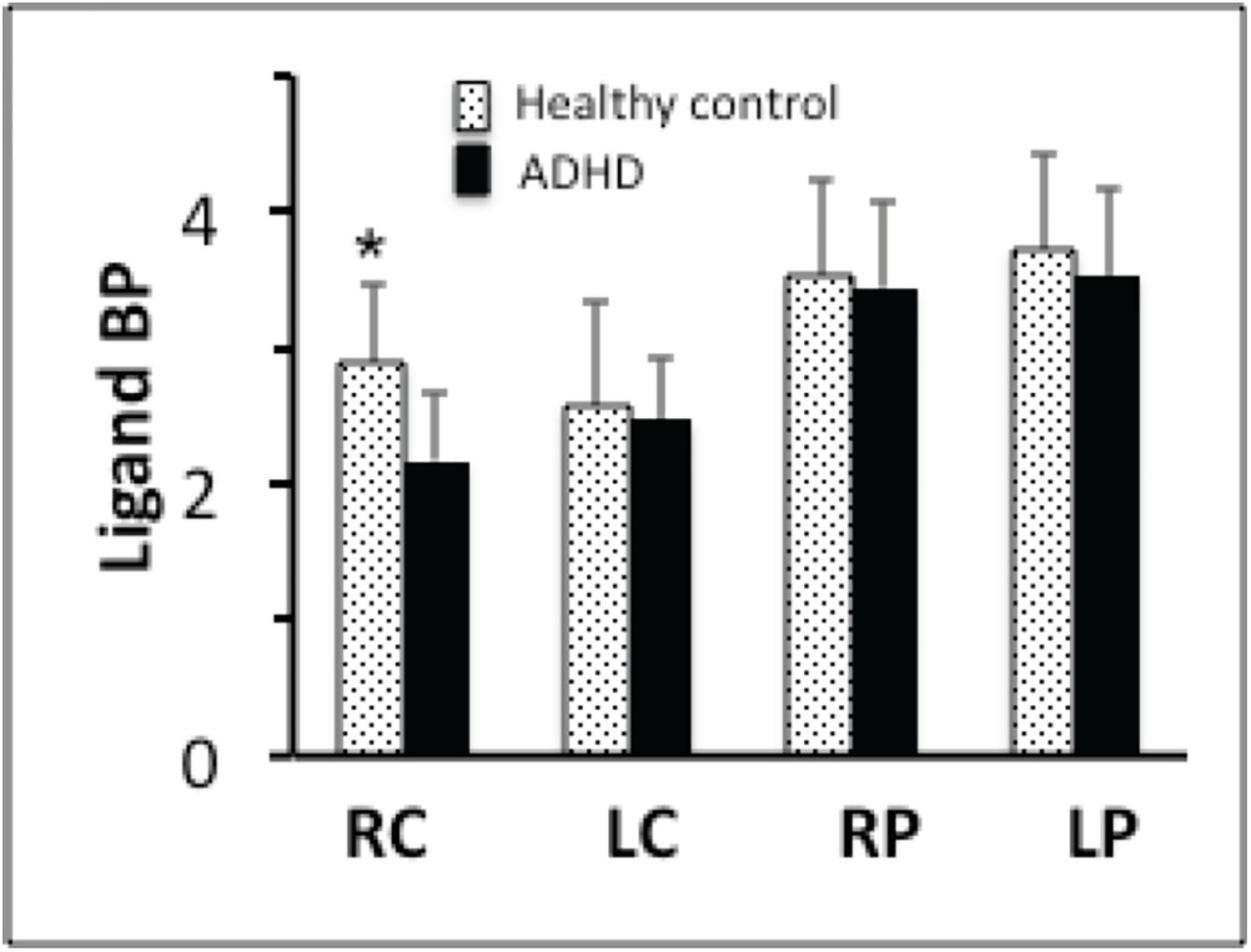
The ligand BP in ADHD and healthy control volunteers in Incongruent condition. ADHD volunteers had lower BP in all striatal areas (suggesting higher amount of dopamine release) but the difference was significant (p = 0.004) only in the right caudate. RC = right caudate, LC = left caudate, RP = right putamen, LP = left putamen.

As mentioned earlier, the PET data were analyzed using LE-SRRM [37] and E-SRTM [38] and values of receptor kinetic parameters were estimated separately in the Congruent and Incongruent condition. To enhance reliability, results obtained in each of the models were reconciled using the criteria developed earlier [27]. Comparison of the ligand BP measured in the Congruent and Incongruent conditions in ADHD volunteers indicated significant reduction in the body of the caudate and middle part of the putamen bilaterally **(Fig 2**) in the Incongruent condition. In addition, there was a significant increase in the rate of ligand displacement from receptor sites in the same areas (**Figs 3 and 4**). Since the ligand BP is proportionally reduced by the amount of endogenously released dopamine and the rate of ligand displacement reflects amount of dopamine released [37, 41], the observation suggests endogenous release of dopamine during task performance (Incongruent condition). In healthy control volunteers reduced BP and increased rate of ligand displacement were observed in the putamen bilaterally and only in the left caudate [27]. The ligand BP measured in the right and left putamen and in the left caudate were similar in the ADHD and healthy control group but in the right caudate it was significantly lower (p = 0.004) in ADHD volunteers (**Figs 5 and 6**). The mean BP was 2.17±0.55 in ADHD and 2.88±0.46 in the healthy control group. Interestingly, stereotactic coordinates of the left caudate and putamen where maximum change in the rate of ligand displacement was observed during task performance were similar in the ADHD and healthy control groups (**Table 2**). The ‘apparent’ rate constant for ligand transfer (min^-1^) from receptor to the plasma (k_2a_) also increased significantly in the right caudate during task performance (Incongruent condition) in the ADHD but not in healthy control group (**Table 3**). Further, the rate constant for ligand transfer (min^-1^) from free to plasma compartment (k_2_) was higher in ADHD as compared to that in healthy control volunteers but the difference was not significant statistically (**Table 4**). Thus, during task performance dopamine was released in the right caudate of only ADHD volunteers. There was no change in the phasic release in the healthy control group (**Figs 5 and 6; Table 2**). Additionally, k_2a_ in the right caudate was significantly higher during task performance in the ADHD but not in healthy control group (**Table 3)** and there was a trend for higher k_2_ in all striatal regions of ADHD volunteers (**Table 4**).

**Table 2:** MNI coordinates the striatal areas where significant changes in the rate of ligand displacement were observed in the Incongruent condition. The table also shows the t-values of the difference in displacement rates observed in the Congruent and Incongruent condition. The data suggests that the locations were almost identical in the ADHD and healthy control volunteers (in the putamen and left caudate) but the changes (t-values) were greater in ADHD.

	ADHD		Healthy Control	
	MNI (x,y,z)	t-value	MNI (x,y,z)	t-value
Right Caudate	12,16,6	4.05	No activation	
Left Caudate	-12,14,6	4.06	-10;14;8	2.58
Right Putamen	26,0,6	4.88	24,4,2	2.10
Left Putamen	-24,0,-6	4.25	-22,4,-6	2.04

**Table 3:** k_2a_ (the ligand washout constant in relation to the total amount of radioactivity in the target tissue) increased significantly in the right caudate in the Incongruent condition in ADHD but not in the healthy control group. NS = non significant.

	Congruent	Incongruent	p; t-value
**ADHD**			
Right Caudate	0.062±0.005	0.069±0.003	0.004; 4.15
Left Caudate	0.065±0.007	0.067±0.004	NS
Right Putamen	0.067±0.003	0.063±0.003	NS
Left Putamen	0.068±0.005	0.065±0.003	NS
**Healthy Control**			
Right Caudate	0.068±0.005	0.068±0.005	NS
Left Caudate	0.067±0.006	0.067±0.004	NS
Right Putamen	0.071±0.006	0.068±0.005	NS
Left Putamen	0.071±0.007	0.069±0.005	NS

**Table 4:** The values of k_2_ (the rate constant for the ligand transfer from free to the plasma compartment) were higher during task performance (Incongruent condition) in the ADHD group but the difference was not significant statistically.

	ADHD		Healthy Control	
	MNI (x,y,z)	k_2_ value	MNI (x,y,z)	k_2_ value
Right Caudate	12,16,6	0.30±0.05	10,16,6	0.29±0.04
Left Caudate	-12,14,6	0.31±0.05	-12,14,6	0.30±0.05
Right Putamen	26,0,6	0.32±0.06	26,0,6	0.31±0.05
Left Putamen	-24,0,-6	0.32±0.06	-22,4,-6	0.30±0.04

## Discussion

Our observation of the reduced tonic and enhanced phasic release of dopamine in the right caudate characterizes the nature of dysregulated dopamine neurotransmission in ADHD. This observation is significant because of the controversy concerning status of dopamine neurotransmission in this condition. Based on indirect evidence previous studies have suggested either increased on decreased dopaminergic activity [3]. Another significant finding of this study is the observation that the dysregulation of dopamine neurotransmission in ADHD is localized in the right caudate. This finding is interesting because the right caudate is critically involved in the processing of cognitive functions that are most affected in ADHD. These functions include executive inhibition and selective attention [42, 43]. Neuroimaging experiments have consistently reported activation of the right frontostriatal circuit (including the right caudate) in experiments that require volunteers to inhibit a response or sustain attention [43]. It is therefore not surprising that a lesion in the right caudate decreases attention span and impairs inhibitory control [44]. Since both of these deficits are important clinical features of ADHD, our finding of localized dysregulation of dopamine neurotransmission is consistent with the functional impairments. It is also consistent with the observation of smaller volume [4, 7] and delayed maturation of fractional anisotropy [45] of the right caudate in ADHD children. It appears that the right caudate is relatively immature and the immaturity is causally related to clinical symptoms. In a recent study it was found that children with smaller right caudate generally score higher in hyperactivity scale and the right caudate volume is strongly correlated to hyperactivity rating in ADHD children [7].

The mechanism, which is responsible for reduced tonic and increased phasic release in the immature right caudate is unclear but our observation of altered values of k_2_, and k_2a_ in ADHD (both at rest and during task performance) indicates that the neurons of right caudate do not have normal receptor-ligand binding kinetics. It could be the reason for altered tonic and phasic release. Another potential reason is increased activity of the dopamine transporters (DAT) in ADHD [46–48]. Since DAT facilitates reuptake of dopamine, an increase in its activity would reduce the tonic pool. Increased DAT activity in ADHD is indicated by the observation of up to 30% higher binding of the DAT ligand in the striatum of these patients [46–48]. Interestingly, the maximum enhancement of DAT activity is observed in the right caudate in the same area where we found decreased tonic and increased phasic release of dopamine. In fact, in a well controlled study, which used a highly selective DAT ligand ^11^C-altropan, significant increase in the ligand binding (17% in males and 22% in females) was observed only in the right caudate of adult ADHD volunteers [48]. Thus, it is possible that increased DAT activity attenuates the tonic release in ADHD and the attenuated tonic pool in turn induces compensatory enhancement of the phasic release because of the reciprocal relationship between the tonic and phasic release [49].

If the primary deficit in ADHD is reduced tonic pool of dopamine due to increased DAT activity, the pharmacological agents that block DAT receptors should restore the deficit and provide clinical relief. It indeed appears to be the case. Thus, methylphenidate which is one of the most effective medications for treatment of ADHD symptoms is also a potent DAT blocker [50]; and levodopa which has no effect on DAT activity does not resolve ADHD symptoms [20]. Our findings therefore suggest that pharmacological treatment of ADHD should focus on raising the tonic pool of dopamine.

Our finding of dysregulated dopamine neurotransmission in the right caudate indicates that clinical symptoms of inattention and impaired response inhibition in ADHD patients are elicited by disrupted processing of the right frontostriatal circuit. It is unclear whether the disruption is caused by reduced tonic release or increased phasic release because the efficiency of dopamine dependent functions is compromised when the neurotransmitter level is either unusually high or low [21, 51].

The results of this study provide the first direct evidence of dysregulated dopamine neurotransmission in ADHD and characterize the nature of dysregulation. Additionally, by showing enhanced phasic and reduced tonic release in the same area (right caudate), the results validate reciprocal relationship between the tonic and phasic release [49]. This evidence will advance our understanding of the nature of normal and dysregulated dopamine neurotransmission in the human brain. The study suggests that relative immaturity of the right caudate is an important neuropathological feature of ADHD and that the pharmacological treatment of ADHD should focus on restoration of attenuated tonic pool of dopamine.
